# Supplementary material for: Patterns of Intron Gain and Loss in Fungi
Source: PLoS Biol. 2004 Nov 30;2(12):e422. doi: 10.1371/journal.pbio.0020422 (PMC532390; doi:10.1371/journal.pbio.0020422)
Supplement: Table S1 — Also available at http://genes.mit.edu/NielsenEtAl/. (4.3 MB ZIP). [file pbio.0020422.st001.zip › NielsenEtAl/html/416.html]

AN7015.1.NCU05523.1.MG00397.1.FG08299.1


```
 CLUSTAL W (1.82) Multiple Sequence Alignments - Introns Inserted


Sequence 1: NCU05523.1	501 aa
Sequence 2: FG08299.1	494 aa
Sequence 3: MG00397.1	490 aa
Sequence 4: AN7015.1	482 aa
Alignment Length: 526 aa
Number Identitical Residues: 164 aa
Alignment Score (without introns) 9172


MG00397.1 	MSQQPVITLSDHLEKQNGATFKKLYQQPATAFAIFRRMLPPLA~KTFVMSLLYMPQPLPL
NCU05523.1	MSLG-GQQLLEYLEKLPGTAFRRLYQSPATALAIFRRMLSYHA1KVIVRGLLYMPDPFPL
FG08299.1 	MSVTPSLQLAEYLEKLPGTTFRKLYQQPSTALAIFRRMLPQLA1KVFVMRILFNPKPILL
AN7015.1  	--MAAASRPFDYLESLPGTVFFKLYQQPSTALAIFRRMLPDLA1KCFVMALLYLKDPLPA
          	    .     ::**.  *:.* :***.*:**:*******.  * * :*  :*:  .*:  

MG00397.1 	TALDSWVKP----~-EAKKNKDQALSILRSMHITTITPVTKEKPVQEMSLTPNFKKSLRL
NCU05523.1	TSLDIWFPPE---~GGGKIHRDRTIAALRSLHILQVSPPSKDR-TQEIQITASFKKGLQL
FG08299.1 	SDLDDWVKP----~-SHKRQKDQALSVMRVLHIVQISTPSKER-PQEMQLTTNFKVSLRL
AN7015.1  	ADLETWVRSESLR2LSFLRERDSALSILGRLHILTNTTTSGNV--RAYMVTDPFAASLRQ
          	: *: *. ..:        .:* ::: :  :**   :. : :   :   :*  *  .*: 

MG00397.1 	ALEGGGSHNSFGVPSSLPIPPQVDVAFLDKWARSRWDAILHYVVNSVEETDSMEPSKKYN
NCU05523.1	AFEGGGEDGSFGVPSSLPVDPSIDIAYLDRYARKKWDDILHYVVNSLGVHGSSEG-----
FG08299.1 	ALSGGGSHNSFGVPSTLQIPPEIDIDFLDRYARKKWEDILHFVVSSVGYKSAGE------
AN7015.1  	ALTGAKETQSFGVLHHIPDDQTVPIHDLDEYARRQWEGVLGYMVGTSGLGIQRD------
          	*: *. .  ****   :     : :  **.:** :*: :* ::*.:       :      

MG00397.1 	FGGSKLQDTVKTLLVQGGLVQRRSSE--RISITKTGFTFLLQEANAQVWTLLLQWLHSVN
NCU05523.1	--VSGPKASVKELLLAGRLVERRPDTRTGIGITQAGFTFLLQESNAQVWTLLLLWLEAAD
FG08299.1 	--SSGPNKSVKELLVAGRLVDRRPSG--AIGITQAGFTFLLQEANAQVWTLLLLWLEAMD
AN7015.1  	---VNLSKGVKQLLQAGHLVEIRDRR---VEITQDGFAFVLQDVGTQVWHILILYVESAE
          	      .  ** **  * **: *      : **: **:*:**: .:*** :*: ::.: :

MG00397.1 	EDNTN--RAVDMLSFLFMLGTLELGQAYDTGALSEERRNMLPDLNDFGLVYIPPSNPDQY
NCU05523.1	HTADSGMDSIDMLSFLFMLSSLELGTAYDTSALTETRRNMLPSLVDFGLIYFPRDTR-QF
FG08299.1 	VNKMAGLEATDMLSFLFVLASMELGRAYDTNALTEQRKNMLPSLVDFGLIYIPNHKRSMF
AN7015.1  	AIGMD---SVEVLSFIFLLSSLELGKSYEKKHLTSNQLRTLTDLADFGIVYQDSPEASHF
          	        : ::***:*:*.::*** :*:.  *:. : . *..* ***::*      . :

MG00397.1 	FPTRLATTLTSGSSALRSVSSGVAAATA----EAGE--------------NNTKGA-IIL
NCU05523.1	FPTRLATTLTSTASALRTVSSAFTAATANPSGEPGTTTSTSGGPAATPDKTQTAAKGIII
FG08299.1 	FPTRLATTLTSSSNSLRSISDGVAAATA-AALQPGQSRAP--GSSATGSSNEQRGS-VII
AN7015.1  	YPTRLATTLTSDSSALSNPISGALSDPD-----GGDS-------------NQPGSGFIII
          	:********** :.:* .  ..  : .       * :             .:  .  :*:

MG00397.1 	ETNFRIYAYTSTPLQIAILALFANLKMRFAGMVTGQLSRHSIKRAISHGITADQIIEYLA
NCU05523.1	ETNYRIYAYTTSPLQIAVLALFCQLNMRFPNMVSGRLTRDSIRQAIGFGITADQIISYLH
FG08299.1 	ETNYRIYAYTQSTLQIAVLALFTKLQMRFPDMVAGRISRQSIRQAINFGITAEQIISYLS
AN7015.1  	ETNYRLYAYTSSPLQISLIALFTTLKYRFPNLVTGKVTRQSIRRAIEMGITADQIISYLA
          	***:*:**** :.***:::***  *: **..:*:*:::*.**::**  ****:***.** 

MG00397.1 	SHAHEQMHRIAAIRNKP--------VLPPTVVDQIRLWQLETERMQVQRGYLFKDFESQA
NCU05523.1	AHAHQQMVREATVTGKP--------VLPPTVVDQIRLWQLENERMQTTAGFLFKDFESTE
FG08299.1 	AHAHDQMRRTASLNNKP--------VLPPTVVDQIRLWQLENERMKTTGGFLFKDFEDHK
AN7015.1  	THAHPQMRKHNVARSTSNQAGMPPSVLPPTVVDQIRLWQLERDRVKATAGFLFKDFVSLA
          	:*** ** :     .....:. ..:**************** :*::.  *:***** .  

MG00397.1 	EFKAIADYADEVGVLIWRSDARQLFFASKTAEIANFIKSEKHRKKLAA
NCU05523.1	EYAALSRYAEEIGVLVWKNDKRQLFFASKHEQLRDYLKSRKKGE----
FG08299.1 	EYMAVAGFAEEVGVLVWRNDVKGMFFASKHEQIRDYLRIRKKAE----
AN7015.1  	EYEAPCRYAEEIGVLIWKSDRKRMFFVTRHEQVAAFLRSRK-------
          	*: * . :*:*:***:*:.* : :**.::  ::  ::: .*
```
